# Comparative Analysis of Different Natural Polymers as Coating Agents for Freeze-Dried Microencapsulation of *Cosmos caudatus* Kunth Compounds

**DOI:** 10.1155/2024/6833341

**Published:** 2024-08-23

**Authors:** Izaz Aqeiluz Zahara, Siti Mariyah Ulfa, Anna Safitri

**Affiliations:** ^1^ Department of Chemistry Faculty of Mathematics and Natural Sciences Brawijaya University, Jl. Veteran, Malang 65145, Indonesia; ^2^ Research Centre of SMONAGENES (Smart Molecules of Natural Genetic Resources) Brawijaya University, Jl. Veteran, Malang 65145, Indonesia

## Abstract

The flavonoid compounds in *C. caudatus* K., known for their various benefits, are prone to quick degradation, leading to reduced biological activity. This research aimed to evaluate the types of coatings: gum Arabic (GA), maltodextrin (MD), and a combination of both (MDGA) in *C. caudatus* K. extract microcapsules. The extract of *C. caudatus* K. was encapsulated by different coating materials, GA, MD, and MDGA, and then dried using a freeze-drying technique. The evaluation was carried out by comparing the encapsulation efficiency values, biological activity, and release tests of each type of microcapsule coating. The research results indicate that coating agents have impacts significantly at *p* < 0.05 on efficiency encapsulation. Flavonoids were retained up to 79.67% by the MDGA coating, compared with 72.8% and 47.66%^a^ retained by single GA and MD coatings, respectively. The results of the encapsulation efficiency are supported by the results of characterization using a scanning electron microscope (SEM), where MDGA has rounder shapes with smoother surfaces compared with a single coating alone, like GA or MD. In addition, by particle size analysis using a particle size analyzer (PSA), the average sizes of MDGA, GA, and MD microcapsules were shown at 154.13 *µ*m, 152 *µ*m, and 166.81 *µ*m, respectively. The three microcapsules showed an order of activities as MDGA > GA > MD coatings in alpha-amylase inhibition assay. Similar results were also shown in the antioxidant assay, which demonstrated that the three microcapsules had moderate antioxidant activities, again in the order of MDGA > GA > MD. The three different coating types showed greater release at pH 7.4 compared to those at pH 2.2 in the controlled release test, which ran from 30 to 120 min. In summary, freeze-drying microencapsulation using biodegradable polymers was identified as a viable method for harnessing the health benefits of *C. caudatus* K. extracts. This process produced a convenient powder form that could be used in drug delivery systems. The use of MDGA mixed coating resulted in better impact based on %EE value and biological activity, as well as improved characteristics of microcapsules compared with single coating.

## 1. Introduction

Research focusing on the use of herbal plants as healing alternatives to synthetic drugs has received significant attention for some time. Herbal plants are considered easier to find and have lower side effects compared to synthetic drugs. The bioactive compounds contained in herbal plants are supposed to be able to cure and prevent disease [1]. One herbal plant with this potential is the *C. caudatus* K. plant, which has various benefits, including antihypertensive, antidiabetic, antioxidant, antiosteoporosis, antifungal, and antibacterial properties [2]. The high content of flavonoid compounds in *C. caudatus* K. can be used as an antidiabetic agent to inhibit the action of the alpha-amylase enzyme [3]. Additionally, its antioxidant activity is equivalent to 100 grams of ascorbic acid, categorizing it as a potent antioxidant [4]. A weakness of using flavonoid compounds in the *C. caudatus* K. plant is their sensitivity to external factors such as temperature, light, oxidation, and pH. In addition, flavonoids are difficult to dissolve in water and have low bioavailability when consumed in the body, leading to suboptimal use [5]. Therefore, special techniques are needed to protect the flavonoid compounds in *C. caudatus* K. plants, one of which is microencapsulation.

Microencapsulation is a technique used to protect bioactive compounds by encapsulating them using a coating, resulting in microsized particles ranging from 5 to 5000 *µ*m [4]. The goal of microencapsulation is to shield active compounds from environmental factors, enhance absorption, and control the release of these compounds in the body [6]. The choice of coating materials is crucial for the success of microcapsules, with maltodextrin (MD) being a commonly used polysaccharide-based biopolymer due to its low viscosity, oxidation protection, and water solubility [5]. However, MD's limited active surface may reduce its emulsifying ability, leading to lower core material stability and encapsulation efficiency [7]. To address this, MD is often combined with gum Arabic (GA), which offers good emulsifying properties and forms protective layers around the core compounds [8, 9]. Gum Arabic's emulsifying ability is attributed to the covalent bonds between heteropolymer sugar-containing protein and carbohydrate chains, enhancing encapsulation efficiency [10]. To preserve volatile flavonoid compounds that are prone to degradation at high temperatures, the freeze-drying method is preferred due to its low-temperature, oxygen-free environment that minimized oxidation [11, 12].

Biological activity testing is essential to determine the biological activity of core compounds after microencapsulation. Some of them are tested for antidiabetic and antioxidant activity. Biological activity tests, such as an antidiabetic test, can be conducted by examining the inhibitory effect of microcapsules on the activity of the alpha-amylase enzyme to assess the biological activity of the core compounds in the microcapsules. The alpha-amylase enzyme converts carbohydrates into glucose. Inhibiting this process can help decrease blood sugar levels. Another biological activity test that can be carried out to determine the potential antioxidant activity of microcapsules is the 2,2-diphenyl-1-picrylhydrazyl (DPPH) method, which is considered practical, effective, fast, and sensitive [13]. In this research, we also want to investigate the release test of the active ingredients in microcapsules, which were carried out in acidic conditions (pH 1–3), representing the condition of the stomach or simulated gastric fluid (SGF), as well as at physiological pH (pH 7.4), representing resistance in the intestine or simulated intestinal fluid (SIF) [14]. The purpose of this study is to determine the differences between three types of coating materials, MD, GA, and MDGA, in microencapsulation processes on *C. caudatus* K. The provision of information on the optimal coating selection strategy to protect the core compounds in the extract of *C. caudatus* K. was deemed very important by this research. Different types of coatings affect the characteristics, morphologies, shapes, and sizes of the microcapsules. The biological activities of the microcapsules in the extract, such as antioxidant and antidiabetic activities, were also affected by the different types of coatings. Therefore, determining the impact of different coatings on the microcapsules of the *C. caudatus* K. extract helped identify the optimal formulation for delivering core compounds in the extract. The aim of this research was to determine the most optimal encapsulation for protecting the core compounds in the extract of *C. caudatus* K., as indicated from encapsulation efficiency calculation, the biological activities of microcapsules, and controlled release tests. Characterization of the microcapsules was performed using Fourier transform infrared (FTIR), particle size analysis (PSA) techniques, and scanning electron microscopy (SEM) techniques to evaluate the effectiveness of the microcapsules produced.

## 2. Materials and Methods

### 2.1. Materials

The research materials used in this study were *C. caudatus* Kunth leaf powder originating from herbal laboratory *Materia Medica* Batu, East Java, Indonesia, 90% ethanol, methanol, acetic acid (pharmaceutical primary standard), NaOH (≥98%, pellets, anhydrous), PBS, HCl, sodium potassium tartrate (≥99%), Na_2_SO_3_, D-(+) glucose (analytical standard), aluminum chloride (≥98%), gum Arabic (from acacia tree, branched polysaccharide), maltodextrin (Sigma-Aldrich, DE 4–7), sodium acetate (Merck, analytical standard 99%), 3,5-dinitrosalicylic acid reagent (DNS) (98%, HPLC grade), soluble starch (potato, ACS grade), alpha-amylase enzyme derived from *Aspergillus oryzae* (Merck, with ≥150 units/mg protein), acarbose (≥95%), 2,2-diphenyl-1-picrylhydrazyl (DPPH reagent) (Merck, analytical standard), quercetin (≥95% HPLC, solid), hydrochloric acid, and ascorbic acid (pharmaceutical secondary standard).

### 2.2. Instrumentation

Several instruments were used including UV-vis spectrophotometer (Shimadzu Model 160A Double Beam), Brookfield viscometer (type DV2T), Fourier transform infrared spectrometer (Shimadzu Prestige 21), scanning electron microscope (TM3000 Hitachi), and particle size analyzer (CILAS 1090).

### 2.3. Extraction Process

A 250 g of powdered *C. caudatus* K. plant was mixed with 1 L of 96% ethanol. The mixture was stirred until dissolved, covered with aluminum foil and a lid, and left to macerate for 3 × 24 h. The filtrate was filtered and evaporated using a rotary evaporator at 68°C and 110 rpm. The extract was further stored at 4°C [15].

### 2.4. Microencapsulation Process

The microencapsulation process of *C. caudatus* K. extracts was performed using three different coating materials, GA, MD, and a combination of GA and MD (MDGA). The microencapsulation process using GA was prepared by dissolving 0.1 g of the extract in 5 mL of ethanol, followed by adding 50 mL of a 4% (w/v) GA solution at pH 5. The extract solution was mixed with the coating solution in 200 mL of distilled water and stirred for 90 min. The colloidal microcapsules were then dried using freeze-drying to obtain microcapsule powder [16].

For microcapsule production using MD as a coating material, 0.1 g of ethanol extract from *C. caudatus* K. was dissolved in 5 mL of methanol. The solution was added to the MD solution prepared by dissolving 0.8 g of MD in 100 mL of acetic acid buffer at pH 5. The mixture was stirred for 90 min until homogenized, and the resulting colloid was dried through freeze-drying to obtain microcapsules.

The use of MDGA as a combination coating for microcapsules of *C. caudatus* K. extract involves weighing 0.1 g of the extract and mixing the extract with 5 mL of ethanol. The coating solution was prepared by dissolving 0.8 g of MD and 1.2 g of GA in 50 mL of acetic acid buffer at pH 5 with a ratio of 2 : 3 (w/v). The extract was combined with the coating solution and added to 200 mL of distilled water. The mixture was homogenized with a magnetic stirrer for 90 min, and the resulting colloid was freeze-dried to obtain microcapsules.

### 2.5. Determination of Total Flavonoid Compound

The determination of flavonoid content began with the preparation of a standard curve of quercetin (at concentrations of 5–20 *µ*g/mL) resulting in the equation (*y* = 0.0392*x*, *R* = 0.9648). Subsequently, 0.1 g of the sample (extract and microcapsules) was dissolved in 2 mL of methanol and incubated at 40°C for 45 min. After incubation, the solution was centrifuged for 10 min. An aliquot of 1.2 mL of the solution was taken and mixed with 1.2 mL of 2% aluminum chloride. The solutions were then mixed and incubated again for 60 min at room temperature. After incubation, the solution was measured for absorbance at the maximum wavelength (420 nm) using a UV-vis spectrophotometer. The absorption value calculated the total flavonoid content in the sample using the following formula [17]:

(1) $$\begin{array}{c} \text{total flavonoid content }\left(\text{TFC}\right)=\frac{\text{sample concentration}\left(\text{mg}/\text{mL}\right)\;x\text{ volume }\left(\text{mL}\right)}{\text{Sample weight }\left(g\right)}. \end{array}$$

### 2.6. Calculation of Encapsulation Efficiency

Encapsulation efficiency (EE) indicates the effectiveness of the coating used to protect the core material (flavonoid compounds). Encapsulation efficiency was calculated by comparing the TFC value obtained from the microcapsules with the extract as shown in the following formula [17]:

(2) $$\begin{array}{c} \text{encapsulation efficiency }\left(\%\right)=\frac{\text{TFC in microcapsules}}{\text{TFC in }C.\;cauda\;tus\;K.\text{ extracts}}\times 100\%. \end{array}$$

### 2.7. Microcapsule Viscosity Test

Microcapsule viscosity was measured using a Brookfield viscometer (type DV2T) with a cylindrical spindle RV-2 at 25°C. The viscosity value was recorded at a rotation speed of 100 rpm in 500 mL microcapsule solution.

### 2.8. Alpha-Amylase Inhibition Assay

In the alpha-amylase enzyme inhibition test, samples of extract, microcapsules, and acarbose were used. Each sample was prepared at concentrations of 20, 40, 60, 80, and 100 *µ*g/mL. 250 *µ*L of each prepared sample was taken at each concentration and mixed with 250 *µ*L of alpha-amylase enzyme (at a concentration of 50 *µ*g/mL). The samples were homogenized and incubated at 37°C for 30 min. After incubation, 250 ml of starch solution (1% w/v) was added to the samples and incubated again at 25°C for 10 min. 500 *µ*L of 3,5-dinitrosalicylic acid (DNS) reagent was added and incubated at 100°C for 5 min until the solution turned brick red. The color-changed solution was then mixed with 5 mL of distilled water and homogenized. The samples were measured using a UV-vis spectrophotometer at a wavelength of 490 nm, and the inhibition was determined using the following formula [18]:

(3) $$\begin{array}{c} \text{inhibition of alpha amylase }\left(\%\right)=\frac{\text{absorbance control}-\text{absorbance sample}}{\text{absorbance control}}\times 100\%. \end{array}$$

The IC_50_ value was determined by creating a linear regression equation where the sample concentration was plotted on the *x*-axis and the percentage of enzyme inhibition on the *y*-axis. Each sample's IC_50_ value was represented by the *y*-value of 50, with the corresponding *x*-value being the IC_50_.

### 2.9. Antioxidant Activity Assay

The samples tested included *C. caudatus* K. extract, microcapsules, and ascorbic acid at different concentrations (extracts: 40–120 *µ*g/mL, microcapsules: 120–200 *µ*g/mL, and ascorbic acid: 2–12 *µ*g/mL). Each sample concentration (3 mL) was mixed with DPPH (50 *µ*g/mL, 2 mL) in a dark room for 20 min. The absorbance was measured at 516 nm using a UV-vis spectrophotometer to calculate the antioxidant activity with the following formula [4]:

(4) $$\begin{array}{c} \text{antioxidant activity }\left(\%\right)=\frac{\text{absorbance control}-\text{absorbance sample}}{\text{absorbance control}}\times 100\%. \end{array}$$

The IC_50_ value was calculated using a method similar to that of the alpha-amylase inhibition assay, with the percentage of antioxidant activity plotted on the *x*-axis and the sample concentration on the *y*-axis.

### 2.10. *In Vitro* Release Assay

Tests were conducted on two types of media: simulated gastric fluid (SGF) and simulated intestinal fluid (SIF). Simulated gastric fluid with a pH of 2.2 was prepared by mixing phosphate-buffered saline with HCl, while SIF with a pH of 7.4 was prepared by adding 5 mL of preconditioned medium at 37°C. The solutions were homogenized using a magnetic stirrer at 100 rpm. Samples were collected at different time intervals of 30, 60, 90, and 120 min. The samples were analyzed using a UV-vis spectrophotometer at a wavelength of 420 nm. The concentration of the released extract was determined as flavonoid content and expressed as a release percentage using the following equation [18]:

(5) $$\begin{array}{c} \text{percentage of release }\left(\%\right)=\frac{\text{TFC released from microcapsules}}{\text{TFC in microcapsules}}\times 100\%. \end{array}$$

### 2.11. Moisture Content

The powder's moisture content was determined gravimetrically by heating 2 g of microcapsules at 105°C until a constant weight had been achieved [19].

### 2.12. Characterization of Microcapsules

Scanning electron microscopy (SEM) was used to analyze the morphology of *C. caudatus* K. extract and microcapsules at magnifications ranging from 9,000 to 15,000x. Fourier transform infrared (FTIR) was employed to identify functional groups in the samples within a wavelength range of 4000–400 cm^−1^. The size distribution of microcapsules was assessed using a particle size analyzer (PSA).

### 2.13. Data Analysis

Data analysis was conducted using SPSS v.26 software. Normality and homogeneity tests were performed on the samples. A one-way ANOVA was conducted with a 95% confidence level (alpha = 0.05). Post hoc Tukey's test was used to determine significant differences, with *p* < 0.05 considered statistically significant [4].

## 3. Results and Discussion

### 3.1. Microencapsulation Process

**Figure 1:**
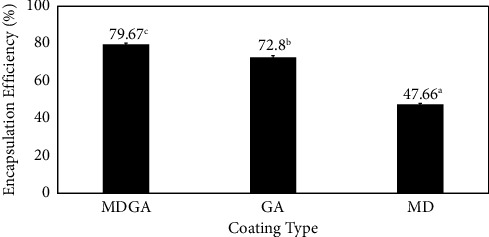
Encapsulation efficiency percent value. The different notations a, b, and c indicate a significant difference at the *α* = 0.05 level.

Figure 1 shows the encapsulation efficiency using three different coating materials. From Figure 1, the EE values using GA and MD coatings only were 72.8% and 47.66%, respectively. The main advantage of GA coating over MD is its ability to form a thick protective layer that shields the core compounds from environmental factors [20]. Gum Arabic is a polysaccharide polymer consisting of three components: arabinogalactan protein, arabinogalactan, and glycoprotein, all of which can act as emulsifiers [21]. The bond between polysaccharides and proteins effectively prevents the decline in flavonoid levels by protecting the compounds due to the complex nature of polyphenols in proteins [22]. However, MD can protect the core compound from oxidation and prevent the protective matrix from cracking, as MD lacks emulsifying properties and cannot be used to protect the base material [7]. The emulsifier properties play a crucial role in determining the viscosity and stability of microcapsules, influencing their encapsulation efficiency (EE). Maltodextrin, with a high saccharide content and low degree of polymerization, contributes to the low EE value [23].

The encapsulation efficiency of the MDGA coating material was shown to be highest at 79.67%. The combination of GA and MD produces superior results due to their complementary properties, which are not present in a single layer. The high viscosity of GA contributes to a high percentage of encapsulation efficiency in the combined coating. This is attributed to GA branched structure with long chains, enabling the formation of a robust coating layer during the drying process. Additionally, the addition of MD enhances the encapsulation quality by preventing matrix cracking [24, 25]. Similar results were observed in the microencapsulation of red chili extract, where the combination of MD and GA exhibited a higher efficiency value of 93.28% compared to a single-layer coating of MD or GA only [19].

Viscosity measurements were conducted as shown in Table 1, indicating that the GA layer exhibited a maximum viscosity of 30.8 cP, while MD had a low viscosity of 13.2 cP. Viscosity plays a crucial role in the emulsification properties of a layer, as viscosity can be adsorbed on the layer surface to protect droplets from flocculation and coalescence [26]. Low viscosity can result in inadequate protection of the core material, leading to less effective layers and the formation of larger droplets, which, in turn, reduces the surface area [27]. However, high viscosity creates a strong protective layer that slows down the diffusion rate of core compounds through the polymer membrane during drying, enhancing core material protection and ultimately increasing encapsulation efficiency [28].

### 3.2. Characterization of Microcapsules with FTIR, SEM, and PSA

**Figure 2:**
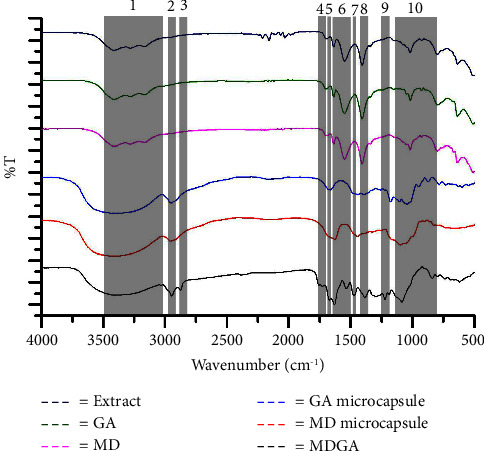
FTIR spectra of *C. caudatus* K. extract, coating materials (MD and GA), and microcapsules prepared in different coating materials.

The FTIR spectroscopy is needed to gather information about the structure and chemical bond characteristics of each substance. The FTIR spectra results determine the effectiveness of the coating in protecting the core material and identify the formation of functional groups in the microcapsules. The analysis was conducted on three types of polymers, revealing characteristic bands as shown in Figure 2 and Table 2. Band 1 indicates the presence of hydrogen molecular bonds from both the coating and the core compound. Additionally, the presence of the OH group is attributed to carboxylic acid and residual water [29]. The characteristic bands for MD and GA production are observed in band 4, within the range of 1639–1737 cm^−1^, indicating vibrations from asymmetric C=O stretching. Bands at 1540–1639 cm^−1^ (band 6) provide information about asymmetric COO stretching of the coating, while COO symmetry is indicated in the absorption band at 1400–1421 cm^−1^. Bands below 1000 cm^−1^ suggest the presence of C-O-C bonds [19].

Upon further examination of the three types of coatings in the spectrum related to hydroxyl O-H, it is apparent that each layer exhibits distinct absorption band values. The spectrum indicates hydrogen bonds formed by the GA and MD layers reacting during the drying process [41]. Absorption in C-O-C bonds provides insights into the characteristics of the bonds present in polysaccharides and core compounds. A comparison of the absorption in the extract reveals that the core material has been coated with a coating material. The emergence of new absorptions from the three types of coatings will result in new spectra [42]. The microcapsules with three coatings (MD, GA, and MDGA) displayed similar absorption behavior, suggesting that all three coatings effectively preserved the chemical structure during freeze-drying. These findings suggest that microcapsules with three different types of polymers can effectively protect the core material [19].

**Figure 3:**
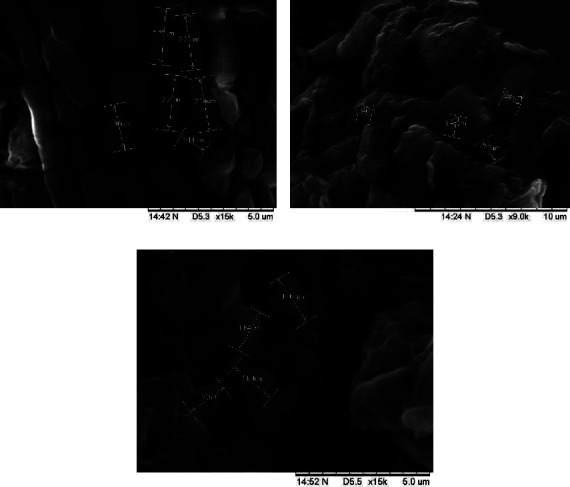
SEM figures of microcapsules prepared in (a) MD coating, (b) GA coating, and (c) MDGA coating.

The SEM analysis was used to examine the shape and surface of microcapsules for any cracks or fractures that could cause degradation and oxidation [43]. Ideally, microcapsules should be uniform, spherical, and have a smooth surface [44]. The morphology of microcapsules in the three types of coatings varied, as shown in Figure 3. The combination of GA and MD resulted in a more rounded shape with a smooth surface and distinct curves. Maltodextrin coating produced microcapsules resembling large, regular chunks with a smooth surface, while GA coating led to flat, irregular lumps with an uneven surface and depressions. The absence of cracks or fragments on the microcapsules' surface indicated effective coating with one of the three types of coatings. Previous studies have shown that MD coating results in a smoother surface due to MD higher molecular weight, leading to a softer surface compared with other coatings [38]. The combined coating of MD and GA resulted in a wrinkled surface due to moisture loss during drying. The hydrophilic nature of the coated core compound can cause the formation of irregular microcapsules that expand and stick together to form a film [19]. Freeze-drying was found to produce microcapsules with a shape resembling broken glass [42]. The chosen drying technique significantly influenced the microencapsulation results. Finer and rounder particles were produced by spray drying, though surface cracks potentially resulted. Conversely, freeze-drying affected the morphology, producing uneven or dented surfaces and more hygroscopic powder due to the low temperature, increasing moisture content [19]. The water content in microcapsules with three different coatings is illustrated in Table 1. Higher moisture content was observed with the addition of gum Arabic, measured at 4.28 ± 0.35%, compared with maltodextrin at 2.14 ± 0.09%. This increase in water content with gum Arabic was attributed to its water-binding capacity within the coating carrier structure. The hydroxyl groups of each coating bound water molecules, resulting in elevated water activity. Increased moisture content in the microcapsules was further contributed to by enhanced adsorption and the higher molecular weight of the coating carrier [45]. A similar profile was observed in the microencapsulation of blackberry powder, where increased water content was shown by the MDGA coating compared with single maltodextrin coating [46]. Higher water content with the addition of gum Arabic was due to its ability to bind water molecules through larger hydrogen bonds formed within the complex heteropolysaccharide structure. Additionally, protein concentration in gum Arabic was increased during the drying process, leading to aggregation during freezing [47]. Consequently, higher water content was exhibited by the GA and MDGA coatings compared to MD, resulting in microcapsules with wrinkles and dents compared to the smooth surface observed with MD.

**Figure 4:**
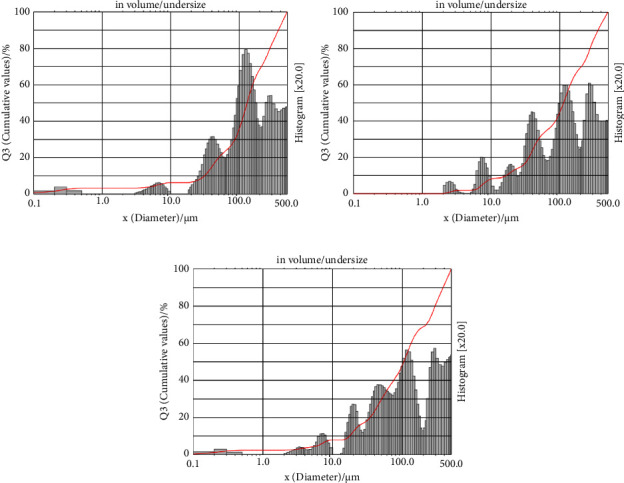
Size distribution using PSA on microcapsules from (a) MD coating, (b) GA coating, and (c) MDGA coating.

Characterization of PSA is important to ensure that the microcapsule size is within the range of 1 to 1000 *µ*m. Results in Table 1 show the particle size distribution for the three types of coatings (MD, GA, and MDGA). Microcapsules with maltodextrin coating are larger compared to MDGA and GA coatings, potentially affecting the stability of microcapsule emulsions. This size difference is due to the droplet size and solution distribution process in the dispersed phase of the maltodextrin coating. A higher maltodextrin ratio results in larger droplet size, leading to decreased microcapsule stability. The weak emulsifying properties of maltodextrin can be improved by incorporating GA, which contains proteins that stabilize emulsions and reduce droplet size, making the microcapsules smaller [48]. Based on Figure 4, peaks are formed in the three types of microcapsules, indicating heterogeneous sizes. Strong peaks are observed in the range of 100–500 *µ*m with intensities >60%, suggesting that the microcapsules are predominantly sized between 100 and 500 *µ*m.

### 3.3. Alpha-Amylase Inhibition Assay

Table 3 presents the results of the alpha-amylase enzyme inhibitory activity test using microcapsules coated with different materials. Microcapsules showed IC_50_ values of 66.50 ± 1.02, 70.27 ± 0.31, and 86.30 ± 1.06 *μ*g/mL when coated with MDGA, GA, and MD, respectively. The addition of GA resulted in a lower IC_50_ value, indicating enhanced alpha-amylase inhibition compared to coatings without GA. Gum Arabic, a polysaccharide composed of arabinose and galactose sugars, is known for its effective inhibition of alpha-amylase and alpha-glucosidase enzymes [49]. This low molecular weight polysaccharide exhibits improved enzyme inhibition due to its structure and reduced transmembrane resistance [50].

In this study, the IC_50_ values of nonmicroencapsulated flavonoid samples and acarbose were calculated as 51.69 and 29.30 *μ*g/mL, respectively. The IC_50_ value of nonmicroencapsulated samples was found to be similar to the inhibition value of flavonoids in microcapsules. The difference in inhibition values can be attributed to the function of acarbose, an oral medication for diabetes mellitus, which may have various side effects when consumed [15]. The decrease in the inhibitory effect of the alpha-amylase enzyme from microcapsules compared to nonencapsulated samples may be due to the slow release and retention of flavonoid compounds in the encapsulation layer. Microencapsulation aims to control the release of compounds in the body rather than increasing inhibitory activity [15, 51].

### 3.4. Antioxidant Activity Assay

Flavonoid compounds found in *C. caudatus* K. extract act as inhibitors of the alpha-amylase enzyme and antioxidants. This study investigated the influence of polymers such as GA and MD on the antioxidant activity of freeze-dried microcapsules.  Table 4 presents the antioxidant activity assessed by the IC_50_ value. The IC_50_ values for microcapsules containing MDGA, GA, and MD were 129.68 *μ*g/mL, 134.54 *μ*g/mL, and 140.67 *μ*g/mL, respectively, indicating a moderate antioxidant activity level (100–150 ppm). Superior antioxidant activity was indicated by lower IC_50_ values. The antioxidant activity values are shown in Table 4, where the highest antioxidant activity was observed in ascorbic acid and its extract, both recognized for their potent antioxidant properties. The primary focus of coating materials was not to enhance antioxidant activity within the core material but to shield the core material from environmental factors. The combination of MDGA coating was found to produce the best IC_50_ results, which was attributable to the protective value of flavonoid compounds within the microcapsules. The high %EE value provided by the combination of coatings indicated enhanced protection for the core compounds. The higher the protective value, the better the flavonoid compounds were preserved within the microcapsules, leading to increased antioxidant activity in neutralizing free radicals and thus preventing damage to the body.

The inclusion of GA enhanced the antioxidant activity of the extract due to its compounds that can combat free radicals and shield against oxidative damage. The use of GA proves to be more advantageous than its absence [34]. The addition of GA enhances antioxidant activity by increasing viscosity and forming a more effective coating layer. The polysaccharide and protein bonds in the GA layer provide better protection against the negative effects of the drying process, stabilizing physical and oxidative impacts to preserve microencapsulated flavonoid compounds [35]. This preservation process aims to improve the ability of flavonoids in microcapsules to capture free radicals [39]. The IC_50_ value profile of this study aligns with other research on microencapsulation of pink peppercorns using GA and MD coatings, showing a 31% increase in DPPH radical inhibition with GA, highlighting its enhanced antioxidant properties [19]. Microencapsulation of eggplant skin with GA and MD layers also demonstrates increased inhibition with the addition of GA, offering superior core material protection compared to a single MD coating due to the unique structure of the coating material wall [29]. However, the resulting microcapsules were adversely impacted by excessively high viscosity, leading to suboptimal antioxidant activity. In this research, optimal protection for the core compounds was provided by employing combined layers, resulting in enhanced antioxidant activity compared with single layers.

### 3.5. *In Vitro* Release Assay

**Figure 5:**
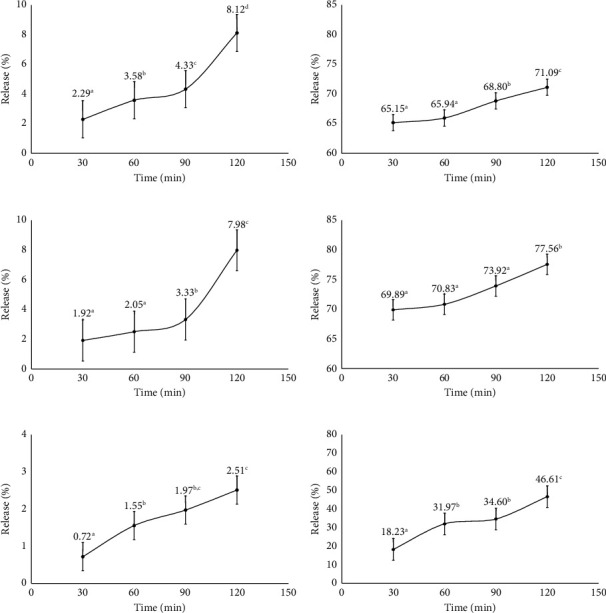
Microcapsule release graph: (a) pH 2.2 MDGA coating, (b) pH 7.4 MDGA coating, (c) pH 2.2 GA coating, (d) pH 7.4 GA coating, (e) pH 2.2 MD coating, and (f) pH 7.4 MD coating. The different notations a, b, and c indicate significant differences at the *α* = 0.05 level.

Figure 5 demonstrates the release of microcapsules over 30 to 120 min at pH 2.2 and 7.4. In the MDGA coating, 2–8% of flavonoids were released at pH 2.2, while at pH 7.4, the release ranged from 65 to 71%. For the GA coating, the release at pH 2.2 was 1–7% and at pH 7.4 was 69–77%. In the single MD coating, the release at pH 2.2 was 0.9–2% and at pH 7.4 was 18–46%. The release profiles in simulated gastric fluid (SGF) for GA and MD coatings show effective protection during the gastric phase. Combining the coating with freeze-drying techniques can enhance encapsulation efficiency and improve protection against active compound release [52]. The maltodextrin coating limits flavonoid release in the stomach at pH 2.2, while the gum Arabic coating allows faster release at pH 7.4, possibly due to interactions between the coating, active compound, and environment. Coating solubility influences the disintegration of the coating material, affecting the release rate of microcapsules. Another study on microcapsules containing vitamin A found that GA exhibited faster release compared with MD or MDGA coatings [53].

**Figure 6:**
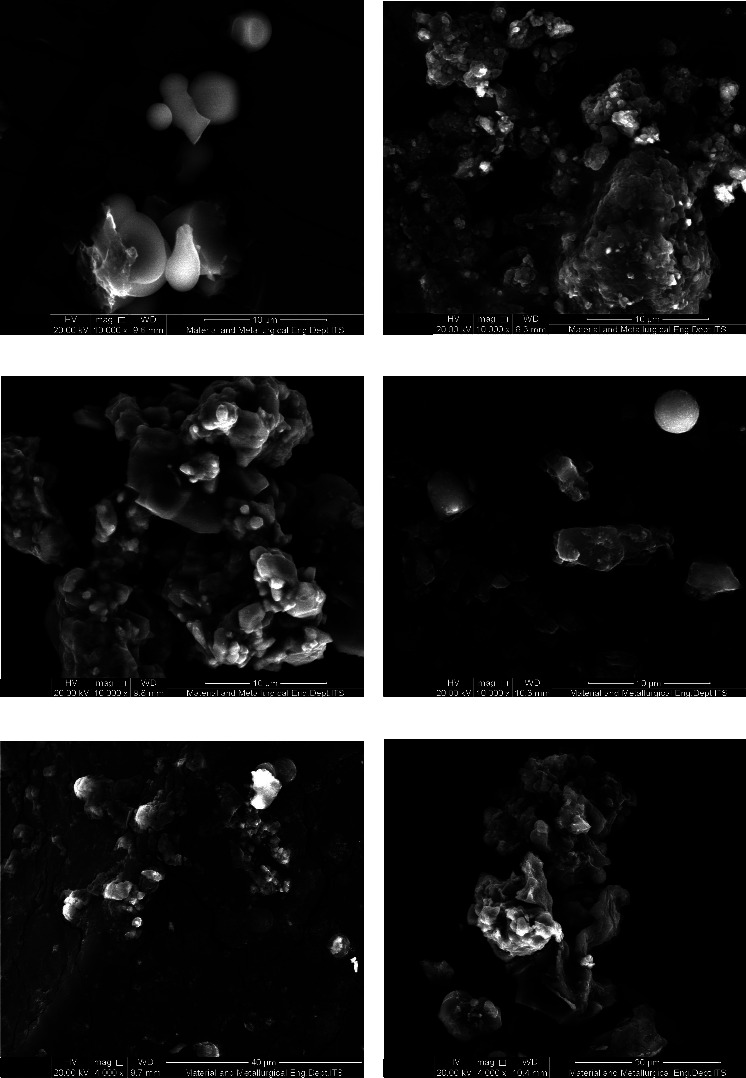
Microcapsule release profile using SEM: (a) pH 2.2 MDGA coating, (b) pH 7.4 MDGA coating, (c) pH 2.2 GA coating, (d) pH 7.4 GA coating, (e) pH 2.2 coating MD, and (f) pH 7.4 MD coating.

SEM analysis of the released images revealed the shapes of the three coatings (MDGA, GA, and MD) postrelease (Figure 6). The maltodextrin coating formed microcapsules with a smooth surface at pH 2.2, indicating resistance to gastric digestion. However, the surface of MD microcapsules at pH 7.4 showed signs of degradation. Both MDGA and GA coatings exhibited surface degradation at pH 2.2, while cracks in GA and MDGA coatings led to core compound release at pH 7.4. The presence of flavonoids in the intestinal environment compromised the stability of the coatings, resulting in increased release. This highlights the protective efficacy of GA or MDGA coatings for flavonoids, especially during the gastric phase.

Gum Arabic has a protein structure with fewer polar hydroxyl groups, providing protection against acids [54]. MD offers better stability for polyphenolic compounds than GA [55]. The prolonged-release effect is due to bioactive diffusion and swelling of the coating material, enhancing protection during the gastric phase [19]. The properties of the coating material, such as solubility, significantly impact the release environment, affecting the release rate from microcapsules [56]. Maltodextrin coating provides better protection in the intestine, while MDGA is more effective in the gastric phase. Microencapsulation aims to protect flavonoids from degradation in the gastric phase, ensuring intact delivery to the intestine. Coating composition can influence the damage to the coating material under gastrointestinal conditions [19]. In this study, the stability and protection of core compounds were more effectively influenced in the acidic phase, such as in the stomach, by the inclusion of GA. Meanwhile, superior protection for core compounds in the intestines was provided by MD coating compared with GA or MDGA coatings.

## 4. Conclusion

Flavonoid compounds in *C. caudatus* K. were effectively protected using microencapsulation with gum Arabic, maltodextrin, and a combination of gum Arabic and maltodextrin. Optimal protection was achieved with the combined coating, yielding the highest encapsulation efficiency (79.67%). FTIR analysis showed that all three microcapsules produced new peaks resembling the absorption spectra of the coatings, indicating effective protection of the core compounds. Scanning electron microscopy analysis revealed that uniform, smooth-surfaced microcapsules resulted from maltodextrin coating, while the addition of gum Arabic caused a dented surface due to moisture loss during drying. PSA results showed an average microcapsule diameter of 152–167 *µ*m. Biological activity as alpha-amylase inhibitors and antioxidants was maintained by the MDGA-coated microcapsules, with IC_50_ values of 66.50 and 129.68 *µ*g/mL, respectively. In the release test, better protection in the stomach phase (pH 2.2) was provided by gum Arabic coating, while more effective protection in the intestinal phase (pH 7.4) was shown by maltodextrin coating. The research highlighted the importance of selecting the right coating material for microencapsulation to preserve core compounds in plant extracts susceptible to environmental degradation. The combination of maltodextrin and gum Arabic achieved a better effect than a single coating. However, this study only compared polysaccharide polymers, indicating that other coating materials and drying techniques beyond freeze-drying should be explored in future research to achieve optimal microcapsules.

## Figures and Tables

**Table 1:** Viscosity, moisture content, and particle size distribution of microcapsules prepared in different coating materials.

No.	Coating material	Viscosity (cP)	Moisture content (%)^∗^	Diameter at 10% (*µ*m)	Diameter at 50% (*µ*m)	Diameter at 90% (*µ*m)	Mean diameter (*µ*m)
1	MDGA	28.4	3.63 ± 0.15^b^	17.50	104.06	384.03	154.13
2	GA	30.8	4.28 ± 0.35^c^	16.62	113.01	358.14	152.00
3	MD	13.2	2.14 ± 0.09^a^	31.09	130.21	373.13	166.81

^∗^The different notations a, b, and c indicate significant differences at the *α* = 0.05 level.

**Table 2:** FTIR spectra assignments for *C. caudatus* K. extract, microcapsules, and coating materials.

Label number	Wavenumber (cm^−1^)						Assignment
	Extracts [30–33]	GA [34–37]	MD [38–40]	GA microcapsules	MD microcapsules	GAMD microcapsules	
1	3385.83	3374.42	3412.92	3411.50–3167.62	3412.92–3006.45	3410.07–3167.62	-O-H stretching
2	2925.16	2935.14	2932.29	—	—	—	-C-H_3_ stretching
3	2853.85	—	—	—	—	—	-C-H_2_ stretching
4	1651–1737	—	1644.42	1697.19	1698.62	1692.91	C=O
5	1610.19	—	1611.62	—	—	—	C=C stretching
6	—	1607.34	1565.98	1637.29–1547.44	1635.86–1547.44	1637.29 1546.01	COO asymmetric stretching
7	1449.03	—	—	—	—	—	-C-H_2_ bending
8	—	1421.93	1420.51	1406.24	1406.24	1406.24	COO symmetric stretching
9	1363.46	—	—	—	—	—	-C-H_3_ bending
10	818.65–1065.38	805.81–1036.86	1032.58–1079.64	925.61–1046.84	925.61–1146.67	925.61–1152.38	C-O-C stretching

**Table 3:** IC_50_ value of *C. caudatus* K. ethanol extract, microcapsules prepared with different coating materials, and acarbose on the inhibition of alpha-amylase activity.

Samples	IC_50_ (*µ*g/mL)^∗^
MDGA	66.50 ± 1.02^c^
GA	70.27 ± 0.31^d^
MD	86.30 ± 1.06^e^
*C. caudatus* K. extract	51.69 ± 0.93^b^
Acarbose	29.30 ± 0.77^a^

^∗^The different notations a, b, c, d, and e indicate significant differences at the *α* = 0.05 level.

**Table 4:** IC_50_ value of *C. caudatus* K. ethanol extract, microcapsules prepared with different coating materials, and ascorbic acid of antioxidant activity assay.

Sample	IC_50_ (*µ*g/mL)^∗^
MDGA	129.68 ± 1.77^c^
GA	134.54 ± 1.52^d,e^
MD	140.67 ± 5.18^e^
*C. caudatus* K. extract	55.76 ± 0.46^b^
Ascorbic acid	4.07 ± 0.14^a^

^∗^The different notations a, b, c, d, and e indicate significant differences at the *α* = 0.05 level.

## Data Availability

The data used to support this study are included in the article and are available from the corresponding author upon request.
